# Economic Burden of Sanfilippo Syndrome in the United States

**DOI:** 10.21203/rs.3.rs-3001450/v1

**Published:** 2023-06-12

**Authors:** Frederick Ashby, Haesuk Park, Mikael Svensson, Coy Heldermon

**Affiliations:** University of Florida College of Medicine; University of Florida College of Pharmacy; University of Florida College of Pharmacy; University of Florida College of Medicine

## Abstract

**Introduction:**

Sanfilippo syndrome is a rare disease and fatal genetic disorder in the United States with no FDA-approved treatment, and no comprehensive assessment of economic disease burden is available.

**Objective:**

To develop a model to estimate the economic burden associated with Sanfilippo syndrome in the United States (US) using valued intangibles (disability-adjusted life years lost) and indirect burden (lost caregiver productivity) from 2023 onward.

**Design and Setting:**

A multistage comorbidity model was generated using publicly available literature on Sanfilippo syndrome disability, and 14 disability weights from the 2010 Global Burden of Disease Study. Attributable increase in caregiver mental health burden and caregiver productivity loss were also estimated using data from the CDC National Comorbidity Survey, retrospective studies on caregiver burden in Sanfilippo syndrome, and the Bureau of Labor Statistics. Monetary valuations were adjusted to USD 2023 and given a 3% discount rate from 2023 onward.

**Main Outcomes and Measures:**

Year-over-year incidence and prevalence of Sanfilippo syndrome was calculated for each age group in each year, and year-over-year disability-adjust life years (DALYs) lost due to patient disability was calculated by comparing to health-adjusted life expectancy (HALE), considering years of life lost (YLLs) due to premature mortality and years lived with disability (YLDs). Intangibles were valued in USD 2023, adjusted for inflation and discounted to provide economic burden of disease.

**Results:**

From 2023–2043, overall economic burden in the US attributable to Sanfilippo syndrome was estimated to be $1.55 billion USD with current standard of care. The burden to individual families exceeded $5.86 million present value from time of birth per child born with Sanfilippo syndrome. These figures are also a conservative estimate, since they do not consider direct cost associated with the disease, as extensive primary data on the direct healthcare cost of Sanfilippo syndrome does not currently exist in the literature.

**Conclusions and Relevance:**

Sanfilippo syndrome is a rare lysosomal storage disease, however the severe burden associated with the disease for individual families demonstrates a profound cumulative impact. Our model represents the first disease burden estimate associated with Sanfilippo syndrome. This underscores the substantial morbidity and mortality burden of Sanfilippo syndrome.

## Introduction

Sanfilippo syndrome (MPS III) is a rare mucopolysaccharidosis within the lysosomal storage disease category, which is characterized by an inability to break down heparan sulfate, a glycosaminoglycan, which leads to accumulation, inflammation, oxidative stress and ultimately the debilitating manifestations of the disease.[1] MPS III is categorized into four subtypes (A, B, C and D) based on which enzyme is affected in the heparan sulfate breakdown pathway,[2] and hence each would require different biological products to replace the defective enzyme. Each subtype has been described with different levels of severity and life-expectancy,[3] yet clinically they remain virtually indistinguishable without molecular testing.[4] Like many rare diseases, there is currently no cure for the MPS III subtypes, however multiple clinical trials have been initiated to attempt enzyme replacement or reduction of heparan sulfate storage.[2, 5, 6] While there are clinical trials for MPS III subtypes A and B, many have failed or been discontinued.[7] Reasons for discontinuation often stem not only from lack of efficacy, but also from perceived small market size, difficulty in designing a trial with a small patient population, lack of clear clinical end-points and challenges demonstrating statistical power and efficacy sufficient for FDA approval in a short time frame.[8–11] Thus, the potential risks to the company performing the trial have historically been seen as incommensurate with the potential financial return, yet trial funding trends in the last few decades have shifted this narrative and demonstrated potential market viability.[12, 13]

Clinical trial costs have historically been a barrier to initiating or continuing clinical trials for rare childhood diseases, considering the risk of failure, and can make investment unappealing.[8–11] Government initiatives in the United States (US), such as the FDA Office of Office of Orphan Products Development, and the NIH Office of Rare Diseases have been formed out the recognition that collectively, these diseases carry great societal burden, and society benefits from public funding to stimulate research and development with the intention of creating marketable products eventually. Despite the perspective that researching orphan diseases is expensive and generally not viable in the market, we found few or no peer-reviewed investigations into what the actual cost of most rare diseases are, particularly MPS III.

Thus, we aimed to estimate the economic burden of MPS III including: (1) the patient intangible health losses expressed through monetary estimates of disability-adjusted life years (DALYs) lost, (2) the caregiver intangible health losses, and (3) economic burden of disease when considering lost productivity of caregivers.

## Methods

### Model Overview and Assumptions

**Figure 1:**
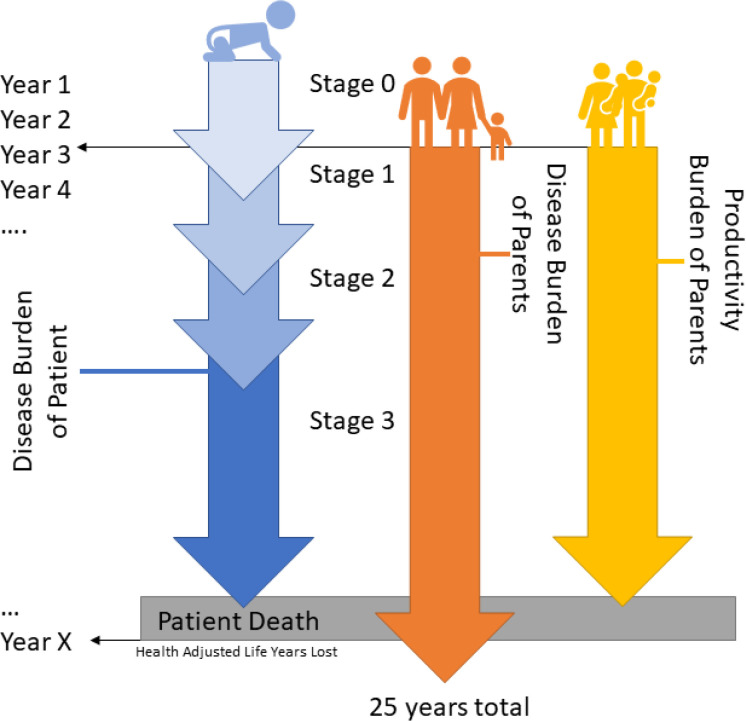
Concept Diagram of Simulated Patient. A conceptual example of a single MPS III patient is shown for the purposes of illustration. Patient burden was calculated by intangibles (DALYs), which included comorbidity after age of onset for each stage of the disease and expected age of death (Year X). The first 2.5 years of life are presumed normal, with no change in disease burden. Economic burden of DALYs for the patient, parents as well as lost productivity for each respective year after onset of symptoms was assigned to their chronologically respective year so discounting rates for the dollar amount could be applied appropriately.

Projected burden of disease estimates were calculated using a model accounting for comorbidity[14] on a multistage scheme for MPS III patients born from 1992–2100 in the US (Fig. 1), presuming only comorbidities associated with MPS III (**Supplementary Table 1**). A total of 1,073 MPS III patients were simulated with a lifelong time horizon. In the same timeframe, 2,146 parents with a time horizon of 25-year after onset of child’s symptoms. Burden of disease considered DALYs lost in the patient due to illness, both parents due to mental health, and lost productivity due to caregiver burden. The value of labor for parents was set to $75,424 annually ($52,378 in wages, $23,046 in social fees, averaged between male and female) based on the US Census and Bureau of Labor Statistics 2019 and it was presumed that the equivalent of one full-time person worth of labor would be lost fulltime during the symptomatic phase of the disease until death of the patient.

We obtained the forecasted birth rate in the US, as calculated by the Institute for Health Metrics and Evaluation (IHME), through GHDx the dataset “Global Fertility, Mortality, Migration, and Population Forecasts 2017–2100.”[15] Due to the unpredicted decline in US fertility rate 2020–2022 compared to the IHME models, this analysis makes the presumption that US fertility rates will normalize to IHME predictions by 2025. The natural sex ratio of male to female live births of 105 to 100 was used for male and female estimate stratifications (UN World Population Prospects 2022). A live-birth prevalence of MPS III in the US of 0.27 per 100,000 live births was used, which was derived from National MPS Society between 1995–2005.[16]

### Disability Adjusted Life Year Modeling: Patients

**Figure 2:**
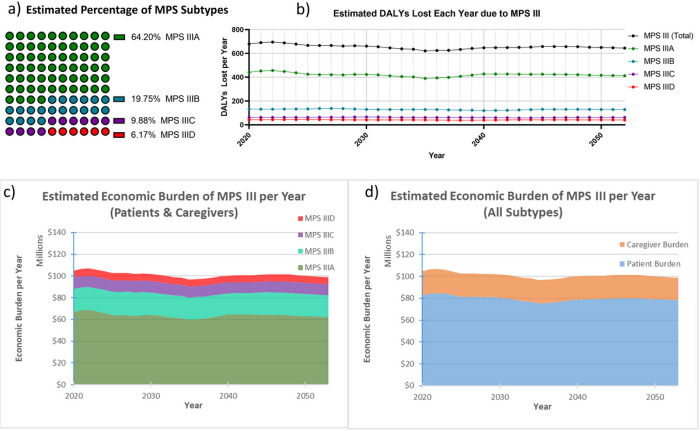
Annual Undiscounted Economic Burden of MPS III in the US. Total undiscounted DALYs within the US are shown above (a) from year 2020–2053, and are based on the IHME projections for the US birthrate, with adjustments from 2020–2025 to account for unpredicted decline in birthrate. These estimates are broken down by MPS III subtype, and presume subtype distribution close to natural rates previously observed in the literature (b). Year-over-year economic burden is shown (c and d) subcategorized by MPS III subtype (c) and patient (DALYs lost) versus caregiver (DALYs lost and lost wages) burden (d). The above figures presume a DALY value of $114,339 in the US, which is the status quo of US healthcare 1996–2016.

Health-adjusted life expectancy at birth for the US was presumed 65.2 for males and 67.0 for females based on the World Health Organization 2022 report.[17] Due to clinical similarity between subtypes,[18] our multi-stage model used the presumption that the average disease course will manifest similarly between subtypes in terms of stage onsets.[1, 4] Disability weight values were derived from the Global Burden of Disease Study 2010.[19] The presumed occurrence of each MPS III subtype was estimated based on the natural rate of each subtype observed in France, the United Kingdom, Greece, and Australia cumulatively: 64.2% MPS IIIA; 19.8% MPS IIIB; 9.9% MPS IIIC; and 6.2% MPS IIID (Fig. 2a).[20, 21]

### Multi-stage Comorbidity Disability Weight Modeling

The average non-adjusted life expectancy or each subtype of MPS III was used: MPS IIIA 15.22 years; MPS IIIB 18.91 years; MPS IIIC 23.43 years.[22] Average life expectancy of MPS IIID was not available, so the weighted average of every other subtype was used (16.86 years) -- this was close to the average lifespan of two reported MPS IIID cases (14 years).[21] The comorbidity calculations for MPS III emulated a three-stage natural history previously described.[1, 4, 22] For stage 1 (onset age 1–4), the presumed average age of onset was the midpoint of the range of onset (2.5 years old), similar with stage 2 (4.0 years old) and stage 3 (11.5 years old). The first 2.5 years of life (Stage 0) were considered healthy for this model,[23, 24] and the final years of life were presumed stage 3 until death. The cumulative disability weight of Stage 0 was 0; Stage 1 was 0.149;[1, 3, 4, 25–28] Stage 2 was 0.357;[1, 4, 25, 27–31] and Stage 3 was 0.68[1, 4, 26–36] (**Supplementary Table 1**).

### Disability Adjusted Life Year Modeling: Caregivers

Caregiver DALYs were factored into caregiver economic burden (**Supplementary Table 2**). For major depressive disorder (MDD) an odds ratio (OR) of 2.90 for mothers and 2.42 for fathers was used.[37] Post-traumatic stress disorder (PTSD) prevalence in parents of MPS III children was presumed to be 26.9% in mothers, and 15.8% in fathers. These statistics reference a retrospective study of MPS III parents in the Netherlands,[37] which was the only available public study on the prevalence of depression, anxiety, and PTSD in caregivers of MPS III patients. We utilized a simple and widely used logistical regression model to obtain estimated risk ratio (eRR)[38] instead of using the OR, which can overestimate the risk, [39–41] and US baseline prevalence of these mental health disorders. The baseline expected US prevalence of clinical depression and general anxiety disorder were presumed to be 10.4% and 19.0% for mothers, and 5.5% and 11.9% in fathers.[42, 43] The baseline expected prevalence of PTSD was presumed to be 5.2% in mothers and 1.8% in fathers based on the National Comorbidity Survey.[44] Because there is very limited data on how long depression, anxiety and PTSD persist in parents of MPS III children, the presumed length of disease was approximated to 25 years after onset of symptoms, regardless of MPS III subtype.

### Disease Burden Estimates

A monetary value per each DALY of $114,339 was used, based on a comprehensive study investigating cost-effectiveness across all US healthcare services from 1996–2016.[45] We used this as our primary analysis since it represents status quo for US healthcare services. A 3% annual discount rate was used accruing from 2023 onward[46]. We also conducted a sensitivity analysis with similar parameters using upper limit of $150,000 and a lower limit of $69,499, since this is reported as an international cost-effectiveness threshold for very-high income countries.[47] A one-time 17% inflation factor was applied to convert USD 2019 to USD 2023 (Bureau of Labor Statistics, 2023).

## Results

### DALYs Lost per MPS III Birth with Current Standard of Care

The multistage model used for this analysis demonstrated an average DALY loss of 55.80, 54.46, 53.10 and 55.08, for a male child born in the US with MPS IIIA, MPS IIIB, MPS IIIC and MPS IIID, respectively. The DALY loss for a female MPS III child born in the US was 58.18, 57.06, 55.70 and 57.68, respectively (**Supplementary Table 3).** Caretaker disease burden for each child born with MPS III, regardless of child sex at birth, was 2.08 DALYs lost for the child’s father and 4.40 DALYs for the child’s mother (**Supplementary Table 3**).

### Estimated Economic Burden of MPS III in the United States

After applying the IHME projections for US birthrate, presuming 0.27 MPS III cases per 100,000 live-birth in the US, a year-over-year estimate of the total number of undiscounted DALYs lost was generated using a dynamic cohort starting at those born in 1992 and ending with those born in 2100 (**Supplementary Fig. 1**). Based on the US birthrate estimates and incidence of MPS III, there are an estimated average of 11 MPS III births each year, and due to the presumed declining US birth rate, this eventually reaches only 8 annual MPS III births by 2100. Based on the weighted average lifespan of MPS III patients, it was also derived that the estimated total US population of MPS III patients may be at a steady state of around 185–195 patients from 2020–2053, with a slow decline that correlates with a declining US birthrate. This timeframe was the focus of the analysis (Fig. 2b). The total estimated undiscounted societal economic burden on an annual basis were also extrapolated and visualized by subtype (Fig. 2c) and patient economic burden compared to caregiver economic burden (Fig. 2d).

### Burden of MPS III Cases 2023–2043

**Figure 3:**
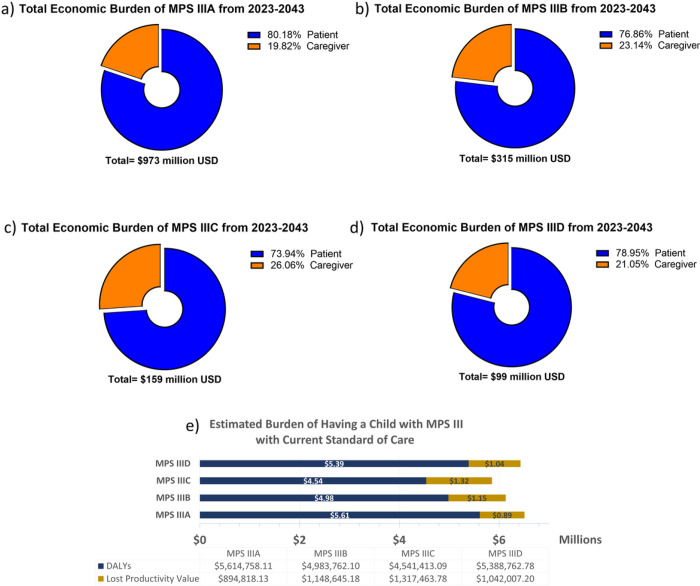
US Societal Burden of MPS III from 2023–2043. The above graphs visualize the presumed disparities between MPS III subtypes regarding discounted burden components (a-d). Overall, all subtypes had between 19.8%–26.1% of their burden composition associated caregivers. When parsing out differences between intangible value lost and lost productivity for parents (d), all subtypes cost families between $0.89m-$1.32m in lost wages alone, and between $4.54m-$5.61m in DALYs lost (both patient and parents).

**Figure 4:**
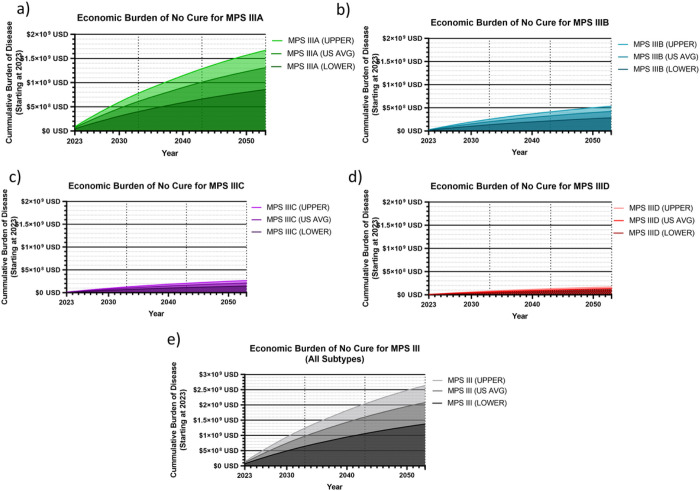
Cumulative Societal Burden of MPS III 2023–2053. The following figures demonstrate the cumulative societal burden of not finding a cure for MPS III by subtype (a-d), starting in 2023 as a zero point, and cumulatively increasing from there. The total cumulative burden for all subtype is also shown (e). The average US healthcare value per DALY of $114,339 was used as the primary analysis, with an upper limit of $150,000 (ICER) and a lower limit of $69,499 was used. An inflation factor of 17% was used to convert to USD 2023 (a-e), and a standard 3% discount rate was used.

The analysis performed for all MPS subtypes demonstrated a cumulative discounted societal burden of $1.55 billion between 2023–2043 alone, with an upper limit of $1.97 billion and a lower limit of $1.02 billion. In total, 79.7% of this amount was from MPS III patient burden, and 21.3% representing caregiver burden. By subtype, burden composition was similar between subtypes: MPS IIIA 19.0% caregiver burden (Fig. 3a); MPS IIIB 22.2% caregiver burden (Fig. 3b); MPS IIIC 24.9% caregiver burden (Fig. 3c); MPS IIID 20.2% caregiver burden (Fig. 3d). Total burden from 2023–2043 by subtype was $973 million for MPS IIIA, $315 million for MPS IIIB, $159 million for MPS IIIC and $99 million for MPS IIID. The total discounted economic burden caused for each individual birth, is $6.50 million for MPS IIIA; $6.13 million for MPS IIIB; $5.86 million for MPS IIIC; and $6.43 million for MPS IIID (Fig. 3f). This was calculated by simulating a single hypothetical patient, discounting from year of birth, and taking into account caregiver burden. Economic burden composition was similar regardless of year, as this analysis presumes no improvement in standard of care for MPS III. For reference, the dynamic present value of damages each subtype of MPS III up until a given year between 2023–2053 is plotted for MPS IIIA (Fig. 4a), MPS IIIB (Fig. 4b), MPS IIIC (Fig. 4c), MPS IIID (Fig. 4d). Cumulative burden for all subtypes from 2023 to a given year until 2053 are graphed in Fig. 4e with an increased y-axis range.

## Discussion

In this paper, publicly available data was analyzed, and our results suggest that MPS III will continuously remain with an economic burden in the US with an estimated $1.55 billion per year between 2023 and 2043 including children’s lives lost, caretaker mental health, and lost wages. Each family who bears a child afflicted with MPS III can expect to lose on average between $0.89 and $1.32 million in lost wages alone and lose between $4.54 and $5.61 million in collective DALYs lost – for both patient and caregiver. This analysis also suggests that the US MPS III patient population may currently be at a steady-state of around 185 patients, which represents 0.97–1.54% of the entire estimated MPS III patient population worldwide,[48] a higher share than we were expecting given this disease is usually thought of as heavily skewed to specific countries with high carrier prevalence.[49] One important finding to our analysis is that the caregiver economic burden was relatively small in comparison to the societal value of DALYs lost in the child (~ 25% of total).

While this analysis focused on MPS III, a rare lysosomal storage disease in the US (0.27 per 100,000 live births), it is important to consider the litany of neglected rare diseases have been estimated to cost our society $966 billion per year according to the Government Accountability Office (GAO).[50] While no one can predict how soon an effective treatment can be developed before a randomized-control trial is done, understanding the value of the disease can help inform policy and funding decisions. For MPS III, the cumulative disease burden without considering direct cost is estimated to be around $100 million (USD 2023) per year for the next few decades based on our analysis. Investing in a cure would potentially be highly valuable considering the burden associated with MPS III, and the current lack of treatment or palliation. The severe burden to each family with an MPS III child is quite substantial to consider, as diseases such as diabetes have a total yearly cost of around $11,700-$13,241 when considering both direct and indirect costs,[51] despite getting substantially more research funding than MPS III. Currently in the US, there is not even newborn screening for MPS III, which by itself may be cost-effective in driving new research forward and prevent the stress of delayed diagnosis.[52–55] One of the biggest hurdles to MPS III clinical trials is simply acquiring enough patients for enrollment, and newborn screening would make this process an order of magnitude simpler.

While our analysis provides a novel perspective on the societal burden of MPS III, there are obvious limitations that should be considered. As with any estimates, these analyses are based on previously reported disease birth-prevalence, and these may be underestimated due to missed or undiagnosed patients. This report also makes presumptions about a consistent disease birth-prevalence and a predictable birth-rate within the US. This analysis did not calculate direct costs associated with the disease, which would increase the overall economic burden and require extensive primary data collection from MPS III families for accurate assessment. Our analysis also did not consider future immigration/emigration from the US, which has the potential to profoundly skew the prevalence of child-bearing heterozygous disease carriers if involving high prevalence regions, such as Europe and the Middle East. We also took a conservative stance in our analysis about the value of lost productivity from the MPS III patients directly, so it is important to note that a functional cure would have implications for marginally increasing the workforce as well.

Regarding our analysis, we emphasize that the combined economic burden of over $5.86 million per case of MPS III born is a representation of the total potential present value of curative treatment from the point of childbirth, not a price target. While complete resolution of the disease is typically the lofty goal of any investigational treatment, future treatments of MPS III will likely recover a fraction of this burden. In the case of MPS III, if treatment plans could recover just 25% of morbidity and mortality associated with the burden of disease for MPS III, it would potentially be collectively valued at tens of millions of dollars per year based on our estimates.

## Conclusions

This estimate is currently, to our knowledge, the only estimate of disease burden associated with Sanfilippo syndrome in the United States, in DALYs as well as valuation of disease burden. We also illustrate novel methods for contextualizing rare disease burden in the US. Policy makers and advocates need to consider the disease burden associated with rare diseases in the context of cumulative damages caused rather than the disease prevalence in isolation, as the later can mask the true theoretical value of funding research in these diseases.

## Data Availability

All data utilized in the manuscript is publicly available as cited. The simulation was performed by Microsoft Excel, and the original work is available from the corresponding author upon reasonable request.

## Units of Measurement

USD 2023: Represents United States dollar accounting for 2023 value.
DALYs: Disability adjusted life years, typically expressed as those lost due to disease.
HALYs: Health adjusted life years, a measure of life expectancy which also considers health.
YLL: Years of life lost, the health adjusted life years lost due to mortality.
YLD: Years lived with disability, the health adjusted life years lost due to morbidity.

## Abbreviations and Symbols

BLS: Bureau of Labor Statistics
CDC: Center for Disease Control and Prevention (United States)
DALY: Disability adjusted life year
eRR: Estimated risk ratio or estimated relative risk
FDA: Food and Drug Administration (United States)
GAO: Government Accountability Office
GHDx: Global health data exchange
HALY: Health adjusted life year
ICER: Institute for Clinical and Economic Review
IHME: Institute for Health Metrics and Evaluation
MDD: Major depressive disorder
MPS: Mucopolysaccharidosis
OR: Odds ratio
PTSD: Post-traumatic stress disorder
RR: Risk ratio or relative risk
UN: United Nations
US: United States
USD: United States Dollar
YLL: Years of life lost
YLD: Years lived with disability
WHO: World Health Organization
$: US dollars
