# Supplementary material for: Economic Burden of Sanfilippo Syndrome in the United States
Source: Res Sq. 2023 Nov 1:rs.3.rs-3001450. Originally published 2023 Jun 12. Preprint. [Version 4] doi: 10.21203/rs.3.rs-3001450/v4 (PMC10312916; doi:10.21203/rs.3.rs-3001450/v4)
Supplement: Supplement 1 [file NIHPPrs3001450v4-supplement-1.pdf]

Tables

Supplementary Table 1: Disability Weights for MPS III Multistage Model			
**Comorbidity (Disability Weight)**	**Stage 1**	**Stage 2**	**Stage 3**
mild developmental delay (0.043)[3, 25, 26]	*		
recurrent ear, nose and throat disease (0.027)[27, 28]	*		
bowel disturbance [mild] (0.074)[1, 4]	*		
moderate hearing loss due to age-related and other hearing loss (0.017) [27, 28]	* (50%)		
hyperactivity (0.045)[25, 29, 30]		*	
sleep disturbances [severe] (0.126)[25, 31]		*	*
bowel disturbance [moderate] (0.188)[1, 4]		*	
moderate developmental delay (0.1)[25]		*	
severe hearing loss due to age-related and other hearing loss (0.158) [4, 27, 28]		*(50%)	
severe loss of intellectual processes and motor function (0.402)[26, 29-31]			*
bowel disturbance [severe] (0.247) [1, 4]			*
severe developmental delay (0.16)[26]			*
complete hearing loss due to age-related and other hearing loss (0.215)[4, 27, 28]			*(50%)
less severe epilepsy (0.263)[32-36]			*(40%)
**Cumulative Disability Weight (Multiplicative Comorbidity Method)**	0.149	0.357	0.698

Supplementary Table 2: Caregiver Risk Increase	Depression		Anxiety		PTSD	
	**OR**	**eRR**	**OR**	**eRR**	**OR**	**eRR**
**Mother**	2.90[37]	2.42	2.42[37]	1.91	5.08[37]	4.19
US Baseline Prevalence	10.4%[42]		19.0% [43]		5.2%[44]	
Absolute Risk Increase for MPS III	14.8%		17.2%		16.6%	
**Father**	2.42[37]	2.25	NS[37]	NS	7.18[37]	6.46
US Baseline Prevalence	5.5%[42]		11.9%[43]		1.8%[44]	
Absolute Risk Increase for MPS III	6.8%		NS		9.8%	

Supplementary Table 3: DALYs per MPS III Birth per Patient	Males Total (YLL, YDL)	Females Total (YLL, YDL)
**MPS IIIA**	55.8 (49.98, 5.50)	58.18 (51.78, 5.50)
**MPS IIIB**	54.46 (46.29, 8.07)	57.06 (48.09, 8.07)
**MPS IIIC**	53.1 (41.77, 11.23)	55.7 (43.57, 11.23)
**MPS IIID**	55.08 (48.34, 6.64)	57.68 (50.14, 6.64)

**DALYs per MPS III Birth (Caregivers)**	**Father**	**Mother**
**All Subtypes**	2.08	4.40

**Supplementary Table 4: Caregiver DALYs by Disease**	**DALYs per MPS III Birth**	
**Comorbidity (Disability Weight)**	**Father**	**Mother**
Moderate major depressive disorder (0.396) [19, 37]	0.678*	1.464*
Moderate Anxiety Disorder (0.133) [19, 37]	N/A	0.572*
PTSD (0.57) [19, 37]	1.401*	2.362*
*Calculations derived from eRR using methods described in main text.		
